# Neuronal growth patterns and synapse formation are mediated by distinct activity-dependent mechanisms

**DOI:** 10.1038/s41598-025-00806-9

**Published:** 2025-05-19

**Authors:** Matthew Yacoub, Fahad Iqbal, Zainab Khan, Atika Syeda, Thomas Lijnse, Naweed I. Syed

**Affiliations:** 1 Hotchkiss Brain Institute, University of Calgary, Calgary, AB T2N 4N1 Canada; 2 Alberta Children’s Hospital Research Institute, University of Calgary, Calgary, AB T2N 4N1 Canada; 3 Department of Cell Biology and Anatomy, University of Calgary, Calgary, AB T2N 4N1 Canada; 4 HHMI Janelia Research Campus, Ashburn, VA 20147 USA; 5 School of Mechanical and Materials Engineering, University College Dublin, Dublin, D04 V1W8 Ireland; 6 UCD Centre for Biomedical Engineering, University College Dublin, Dublin, D04 V1W8 Ireland; 7 Cumming School of Medicine, University of Calgary, 3330-Hospital Drive, NW, Calgary, AB T2N 4N1 Canada

**Keywords:** Axon and dendritic guidance, Neuroscience, Cellular neuroscience

## Abstract

All brain functions in animals rely upon neuronal connectivity that is established during early development. Although the activity-dependent mechanisms are deemed important for brain development and adult synaptic plasticity, the precise cellular and molecular mechanisms remain however, largely unknown. This lack of fundamental knowledge regarding developmental neuronal assembly owes its existence to the complexity of the mammalian brain as cell-cell interactions between individual neurons cannot be investigated directly. Here, we used individually identified synaptic partners from *Lymnaea stagnalis* to interrogate the role of neuronal activity patterns over an extended time period during various growth time points and synaptogenesis. Using intracellular recordings, microelectrode arrays, and time-lapse imaging, we identified unique patterns of activity throughout neurite outgrowth and synapse formation. Perturbation of voltage-gated Ca^2+^ channels compromised neuronal growth patterns which also invoked a protein kinase A mediated pathway. Our findings underscore the importance of unique activity patterns in regulating neuronal growth, neurite branching, and synapse formation, and identify the underlying cellular and molecular mechanisms.

## Introduction

The mammalian brain is comprised of a complex system of neuronal networks which work together to process information and enable a wide range of behaviors throughout life^1^. Along with various cellular and molecular cascades, activity-dependent mechanisms have been implicated in the formation of synapses and synaptic plasticity. For example, blocking activity either directly or indirectly by preventing sensory input to the visual cortex results in the loss of function ascribed to those networks^2^. This suggests that neuronal electrical activity plays an important key role in ensuring proper wiring of the brain during critical periods of development^3^. The neuronal growth patterns and connections established during development are however not static rather continuously refined throughout life, invoking synaptic plasticity which forms the basis for learning and memory^4^. This connectivity in turn relies in myriad ways on activity exhibited by most neurons even prior to establishing physical contacts with their putative partners^5^. However, the precise role of neuronal activity in neurite initiation, outgrowth, branching, and synapse formation remains to be fully defined.

Various vertebrate models have been leveraged to investigate the role of neuronal activity in defining growth patterns of neurons. For instance, Yamada et al. used thalamocortical (TC) neurons in organotypic cultures and genetically manipulated their Kir2.1 potassium channels to demonstrate that both pre- and postsynaptic activity was required for axonal/dendritic branching^6^. Using this same TC system, another research group demonstrated that blocking both inwardly rectifying potassium channels Kir2.1 and bacterial voltage-gated sodium channel (NaChBac), suppressed activity while reducing both axon branching and synapse formation^7^. However, identifying specific activity patterns that function either to suppress or promote initial growth at the level of single neurons remains to be determined. Fields et al. on the other hand, stimulated dorsal root ganglion neurons from mice and demonstrated that electrical activity could prevent neurite outgrowth when monitored at the filopodial level^8^. Whereas this study provided evidence that electrical activity may regulate neurite outgrowth, the precise patterns of activity and the underlying cellular mechanisms remain undefined.

Neuronal activity has also been shown in other models to regulate axon branching through downstream calcium (Ca^2+^) influx mediated via various Ca^2+^ channels^9^. For instance, the bundles of peripheral axons have been observed to exhibit synchronized waves of Ca^2+^ oscillations which were proposed to trigger gene induction for proteins required at various neurodevelopmental stages^10^. Whether these Ca^2+^ waves resulted from spontaneous neuronal or axonal discharges was not however investigated. Some other studies have shown that triggering action potentials inhibits neurite outgrowth and causes growth cone collapse in cultured snail neurons^11^. Similarly, activity-induced increase in intracellular [Ca^2+^] was shown to inhibit neuronal outgrowth in vertebrate neurons^12^. Based on these studies, Kater and Mills proposed the “Ca^2+^-window” hypothesis where intracellular Ca^2+^ concentration was required to stay within a prescribed range, and any fluctuations either below or above the window range was deemed detrimental to neurite outgrowth^13^. This model did not, however, identify how spontaneous activity and the ensuing Ca^2+^ homeostasis could regulate the precise patterns of growth at various stages of neuronal development.

Developing neurons are endowed with both neurotransmitter secretory and responsive machinery even prior to contact with their synaptic partners^14-16^. The neurotransmitter release from developing neurons is thought to serve as diffusible signals which may regulate growth of neighboring neurons either in a permissive or suppressive manner^17-20^. Inhibiting serotonin-receptor interactions during development for instance, was shown to result in aberrant neuronal projections^21^, whereas blocking serotonin release has been shown to decrease the synaptic strength^22^. A direct interaction and interdependence between electrical activity, axonal growth, growth cone branching, and synapse formation under physiological conditions has not however been demonstrated.

The *Lymnaea stagnalis* model has been used extensively to characterize fundamental mechanisms governing neuronal development and synaptic connectivity both in vivo and *in vitro at the level of individual neurons*,* growth cones and growth balls*^20,23-25^. One of the well-defined synaptic partners in this model is the postsynaptic left pedal dorsal 1 (LPeD1) and its presynaptic partner visceral dorsal 4 (VD4) which comprise the cardiorespiratory circuit of the mollusk. These synaptic partners have previously been shown to recapitulate their in vivo growth and synaptic connectivity *in* vitro^24^ in a manner analogous to that of an intact animal. LPeD1 is a serotonergic neuron with which its presynaptic partner VD4, forms a cholinergic synapse. Notwithstanding the fact these adult *Lymnaea* neurons recapitulate their developmental patterns of connectivity in vitro in the presence of brain conditioned medium, and exhibit spontaneous activity, the precise interplay between neuronal activity, their growth patterns and synaptogenesis remains largely unknown. Here we asked the question whether neuronal activity patterns differ at various growth stages including both during and after synapse formation, and if physiological and pharmacological perturbation of this activity would compromise their innate patterns of growth. We next sought to define the underlying mechanisms involving activity-dependent calcium influx and PKA mediated signaling.

## Results

### Neuritic branching patterns may be influenced by number of spikes in a burst

**Fig. 1:**
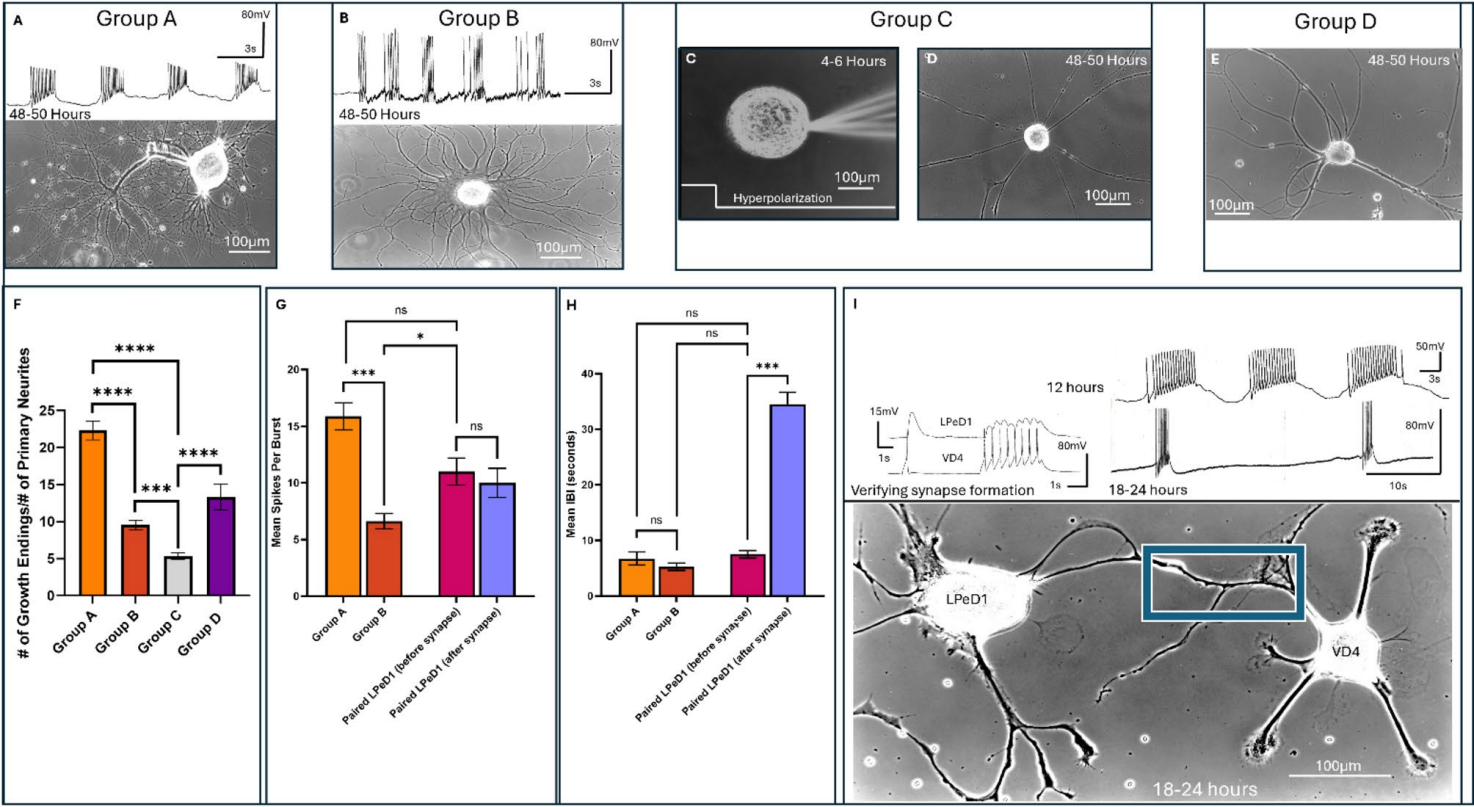
Endogenous neuronal activity patterns are correlated with distinct growth patterns in paired and unpaired LPeD1 neurons. **(A)** An unpaired LPeD1 neuron exhibiting bursts consisting of greater number of spikes concomitant with extensive growth (Group A, *n* = 8) (recorded at 48–50 h). **(B)** Spontaneous bursting consisting of fewer spikes in unpaired LPeD1 neurons, which exhibited a growth pattern most commonly observed in LPeD1 neurons (Group B, *n* = 8) (recorded at 48–50 h). **(C)** Unpaired LPeD1 neurons were hyperpolarized with 0.2-0.3nA current prior to growth initiation (at 4–6 h) (Group C, *n* = 10). **(D)** Unpaired LPeD1 neurons that were hyperpolarized at 4–6 h post-plating (prior to exhibiting growth) and examined at 48–50 h post-plating, exhibited thinner neurites which often remained unbranched. **(E)** Unpaired LPeD1 neurons were impaled with an electrode (at 4–6 h and recordings maintained for 48–50 h) prior to growth initiation (Group D, *n* = 12) but not hyperpolarized. **(F)** Ratio of growth endings to primary neurites from unpaired LPeD1 neurons following 48-hours of growth. **(G)** Mean spikes per burst and **(H)** IBI for endogenous activity from paired and unpaired LPeD1 neurons. **(I)** Paired LPeD1 neuronal activity patterns before (12-hours) and after synapse formation (18–24 h) with VD4 (*n* = 6). The blue box indicates where the synapse likely formed, and recordings from VD4 (pre-synaptic) and LPeD1 (post-synaptic) verified synapse formation with 1:1 excitatory post-synaptic potentials (EPSPs). **Statistics: (F-H)** used a one-way ANOVA (ratio of growth endings to primary neurites after 48-hours: F[3, 34] = 29.43 (*P* < 0.0001), Spikes per Burst: F[2, 19] = 22.17 (*P <* 0.0001), IBI: F[2, 19] = 1.535 (*P* = 0.2411) followed by Tukey’s multiple comparisons test. A paired t-test was used to compare LPeD1 neurons cultured with VD4 before and after synapse formation for spikes per burst [t = 1.732, df = 5, *P* = 0.2876] and IBI [t = 13.07, df = 5, *P* = 0.0004]. Holm-Bonferroni correction was applied to account for family-wise error rate. Error-bars represent SEM, all comparison tests were two-tailed. n.s *p* > 0.05, *p < 0.05, ***p < 0.001, ****p < 0.0001.

We first sought to define various patterns of spontaneous neuronal activity and how they might correspond with distinct growth patterns in individually identified *Lymnaea* neurons. LPeD1 neurons were individually isolated and plated in the presence Brain Conditioned Medium (CM). Generally, neurons began sprouting within 6–8 h after plating onto the poly-L-Lysine substrate. Neurons were then impaled with intracellular electrodes to record spontaneous activity - concomitant with time-lapse recordings of their growth patterns **(**Fig. 1A-B**)**. While we occasionally observed some tonic activity (single spikes), neurons consistently exhibited a bursting pattern. It was interesting to note that the neurons with more synchronized and robust patterns of spontaneous bursting (Group A, ≥ 10 spikes per burst) exhibited elaborate branching patterns, albeit with thinner and finer neurites **(**Fig. 1A**)**. In contrast, neurons that exhibited bursts consisting of fewer spikes (Group B, < 10 spikes per burst) which were often accompanied by discernable individual action potentials corresponded with less elaborate branching **(**Fig. 1B**)**. It is important to note that since individual sprouts were often difficult to discern in all instances, we opted to focus only on those neurites that had emanated from the cell body directly. Moreover, in all the experiments, a neuron was considered to have sprouted if the length of its primary neurites extended five soma diameter^26,27^. Specifically, in instances where extensive neuronal branching had occurred, we noted that the bursts appeared longer and consisted of more spikes per burst (*n* = 8 neurons). When quantified, we found that in instances where more elaborate branching had occurred **(**Fig. 1A**)**, the number of spikes per burst (15.88 ± 3.357) were greater than those neurons that exhibited less extensive branching (*n* = 8, 6.625 ± 1.923, *p* = 0.0002). However, when the inter-burst intervals (IBI), between the two groups were compared, we did not find significant differences between the neurons exhibiting more elaborate branching patterns (6.750 ± 3.327) as compared to those with fewer total neurites and finer branching patterns (5.250 ± 1.909, *p* > 0.9999). Taken together, these data demonstrate that there does indeed exist, at the least, an association between spontaneous neuronal firing patterns and the extent of finer branches growth in individually identified neurons. These differences were, however, subtle in terms of frequency, with there being no significant difference in their IBIs, but the spikes per burst nevertheless exhibited a significant change. Taken together, the above data underscore that specific, spontaneous neuronal firing patterns of neurons may underly their unique growth patterns.

## Blocking neuronal activity perturbs branching

We next asked the question whether blocking electrical activity physiologically would perturb neuronal growth patterns in single cells. To test this postulate, we manipulated neuronal activity by direct intracellular current injections. Specifically, LPeD1 neurons were impaled with sharp electrodes during various growth phases **(**Fig. 1C**)** after 4–6 h of plating, and prior to them exhibiting growth (Group C). We tested for the resting membrane potential (-50-60mV) and triggered individual actions potentials to confirm that indeed a cell had been impaled appropriately. Hyperpolarizing current (0.2-0.3nA) was injected to prevent spontaneous activity in all neurons (*n* = 10). Under such experimental conditions, we noted that whereas all neurons exhibited growth, they were, however primarily devoid of branching. Moreover, the sprouted neurons had fewer and much thinner branches which emanated directly from the cell body **(**Fig. 1D**)**. The data from these hyperpolarized neurons was compared with a control condition where unpaired LPeD1 neurons had a sharp electrode inserted at 4–6 h following plating as above and their electrophysiological parameters confirmed but the membrane was not hyperpolarized **(**Fig. 1E, **Group D)**. A ratio of the number of neurite tips (growth/growth cone endings) to the number of primary neurites was then used to deduce the extent of total neuritic branching **(**Fig. 1F**)**. Using this metric we found that there was significantly greater branching in Group A (22.30 ± 3.5322, *p* < 0.0001) and Group B neurons (9.55 ± 1.757, *p* = 0.0004) as compared to Group C (5.337 ± 1.375). Moreover, Group A neurons which had exhibited bursts consisting of more spikes also had significantly more extensive branching compared to Group B (*p* < 0.0001). The extent of branching present after 48 h post-plating in Group D (13.30 ± 6.008) was significantly greater than in Group C (*p* < 0.0001). These data thus affirmed that blocking neuronal activity during active growth phase significantly alters their growth patterns. These data demonstrate that while preventing electrical activity in the neurons does not completely block neuronal sprouting, it does nevertheless significantly affect their branching pattern. Altogether, these findings **(**Fig. 1A-H**)** further establish the importance of spontaneous neuronal activity in regulating the extent of overall growth and neuritic branching.

## Burst frequency decreases following synapse formation

We next sought to determine whether the electrical activity patterns of LPeD1 neurons were altered following synaptic contact with its presynaptic partner, VD4. For these experiments, we only focused on those neurons exhibiting activity patterns that were like those shown in Fig. 1B (Group B), while making concurrent intracellular recordings from the paired neurons. Specifically, neurons were paired in close proximity (to ensure proximity of synaptic contact points with the recording site (i.e. somata since the efficacy of recorded synaptic strength differs if the synapses are formed between distantly contacting branches), and continuous intracellular recordings were made after 6 h of plating. We often noted that within 20–30 min of neuronal physical contact (typically around 18 h), induced action potentials in VD4 generated 1:1 excitatory postsynaptic potentials in LPeD1 **(**Fig. 1I**)**, whereas, triggering bursts produced compound postsynaptic potentials (PSPs) (*n* = 6). This confirmed that a synaptic contact was indeed established. We next asked whether synaptic connectivity impacted neuronal activity patterns at the level of individual neurons after synapse formation. It was interesting to note that after the establishment of a synaptic connection, the average number of spikes within a burst exhibited by LPeD1 did not change significantly (from 11 ± 2.898 to 10 ± 3.162, *p* = 0.2876), however, the IBI was prolonged significantly (from 7.50 ± 1.643s to 34.50 ± 5.244s, *p* = 0.0004). It was also noteworthy that as compared with individually plated neurons, the firing patterns of neurons when co-cultured with their respective synaptic partners were significantly different. We were surprised to find that even prior to synapse formation, paired neurons (*n* = 6) exhibited increased spikes per burst (11 ± 2.898 vs. 6.625 ± 1.923, *p* = 0.0365) compared to unpaired LPeD1 neurons in Group B (*n* = 8). This may indicate that the presence of a presynaptic partner could somehow influence the firing patterns of the postsynaptic neuron when paired in closer proximity. Taken together, these data demonstrate that; (a) LPeD1 and VD4 recapitulate their patterns of synapse formation within hours of contacts, (b) the spontaneous neuronal activity patterns of paired neurons, when platted in close proximity are different than those of the single neurons, (c) activity patterns of LPeD1 change within minutes to hours of synaptic connectivity, the most significant change being in the inter-burst intervals. These data are summarized in Fig. 1G-I.

## Neuronal burst generation in a physiological range triggers neuritic branching

**Fig. 2:**
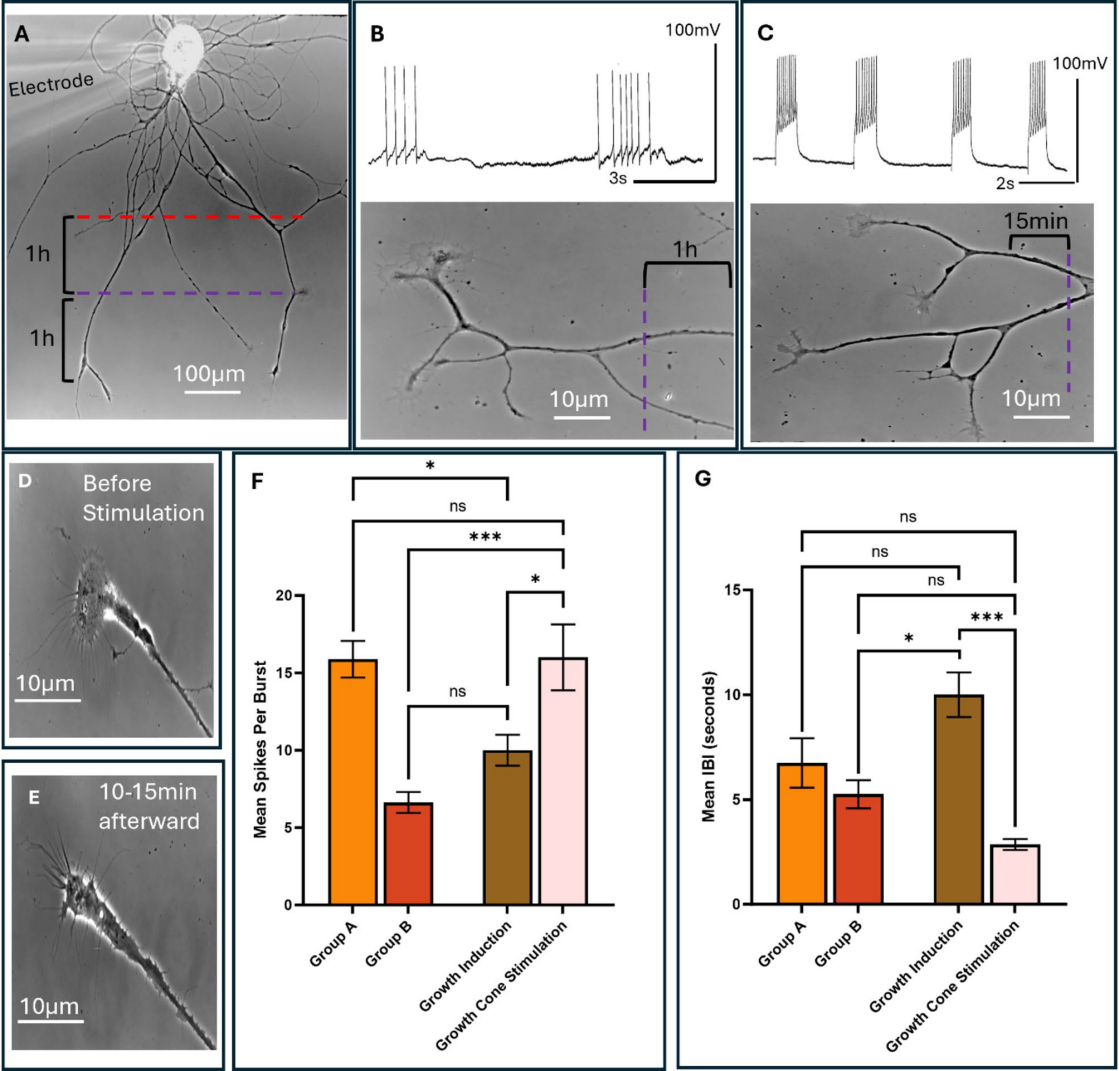
Perturbations of electrical activity in LPeD1 neurons via direct intracellular current injection influence growth and axonal branching. (**A**) Shows an unpaired LPeD1 neuron being impaled with an intracellular electrode at (30–36 h) when the neuron had exhibited growth and branching. LPeD1 was hyperpolarized (0.2-0.3nA) for 1 h, the starting point indicated at the red line and was subsequently released from hyperpolarization at the purple line. **(B)** Shows an image of another LPeD1 neuron that was released from hyperpolarization (purple line). Within 30 min to 1–2 h of resumption of endogenous bursting, this triggered subsequent branching (*n* = 12). **(C)** Shows another LPeD1 neuron where following the release of hyperpolarization, bursting activity was triggered by injecting 0.1-0.2nA depolarizing current (purple line) which was followed by the resumption of neuritic/growth cone branching within 1 (*n* = 6). **(D)** LPeD1 growth cone prior to triggering prolonged bursts for action potentials (3–5 s) over a period of 5–10 min. **(E)** Same LPeD1 growth cone collapsed followed by neurite retraction within 10–15 min (*n* = 7). **(F)** Spikes per burst of unpaired LPeD1 neurons during endogenous (Groups A and B) and triggered bursting activity. **(G)** IBI of unpaired LPeD1 neurons during endogenous (Groups A and B) and triggered bursts. **Statistics**: **(F)** and **(G)** used one-way ANOVA (for spikes per burst: F[3, 25] = 12.49 (*P* < 0.0001), for IBI: F[3, 25] = 10.37 (*P* = 0.0001) followed by Tukey’s multiple comparisons test. A Holm-Bonferroni correction was applied to all p-values to account for family-wise error rate. Error bars indicate SEM, all comparison tests were two-tailed. n.s *p* > 0.05, *p < 0.05, **p < 0.01, and ***p < 0.001. A scale bar is provided for all images.

Having observed that endogenous LPeD1 bursting corresponded with neuritic branching, we next sought to determine if these morphological changes could be mimicked through exogenous stimulation. After neuronal sprouting **(**Fig. 2A**)**, LPeD1 neurons were hyperpolarized for 1–2 h while constantly monitoring their growth patterns. We discovered that after 30 min of hyperpolarizing the neurons (0.2-0.3nA current, red line), they stopped exhibiting branching when monitored at the level of single neurites/growth cone (i.e. they continued to extend, albeit unbranched). We then allowed the neurons to either exhibit spontaneous bursts by releasing them from hyperpolarization **(**Fig. 2B**)** or mimicked those activity patterns with direct intracellular depolarizations (0.1-0.2nA - Fig. 2C). When LPeD1 neurons were released from hyperpolarization and allowed to exhibit spontaneous activity (purple line in Fig. 2A-B), we observed that the individually monitored neurites/growth cones began branching within 30 min to 1–2 h following the resumption of activity (Fig. 2B, *n* = 12). We then asked whether mimicking the spontaneous patterns via direct intracellular depolarizations would also suffice to trigger growth cone branching. Indeed, consistent with spontaneous activity, direct stimulation of LPeD1 neurons for a period of 20–30 s (four to five bursts), also triggered growth cone branching within 15 min (purple line in Fig. 2C) in previously hyperpolarized neurons (*n* = 6). It is important to note that greater stimulation intensity with short pulses was deemed essential as the neurons had already extended extensive branches that were at a distant from the recording sites, and that the injected current would have otherwise spread throughout the sprouted cell. We next asked whether exceeding the growth permissive activity pattern would adversely affect the branching patterns of individual growth cones. To test this postulate, we triggered either one or two intense bursts of depolarization (0.3-0.4nA), lasting 5–10 s. Such depolarizing pulses triggered growth cone collapse within 10–15 min and the retraction of neurites in all instances of the stimulated neurons (Fig. 2D-E, *n* = 7). When quantified and compared to the stimulation required to induce branching (growth induction, a significantly higher number of spikes per burst was required to directly cause growth cone collapse as mentioned above (16.00 ± 5.627 vs. 10.00 ± 2.449, *p* = 0.0365), and a much shorter IBI (2.857 ± 0.6901 vs. 10.00 ± 2.608, *p* < 0.0005) (Fig. 2F-G). Taken together, these data demonstrate a direct relationship between neuronal bursting activity and their growth patterns.

We then sought to compare the extent to which the mean bursting parameters of our stimulation paradigm were consistent with those of the endogenous LPeD1 bursting underlying neuritic branching. We found that an average of 10 $\:\pm\:$ 2.5 spikes/burst was required during stimulation to induce growth/branching, which did not present significant differences as compared to observed Groups A and B endogenous activity (Fig. 2F, *p* = 0.2858). Moreover, there were no significant differences between the average stimulation IBI to induce branching and that seen during endogenous growth in Group A (Fig. 2G, *p* = 0.1146). Taken together, the above data highlights the importance of various distinct patterns of electrical activity and how they evoke unique growth patterns.

*Bursting activity triggers Calcium influx thoughout the neuron.*

**Fig. 3:**
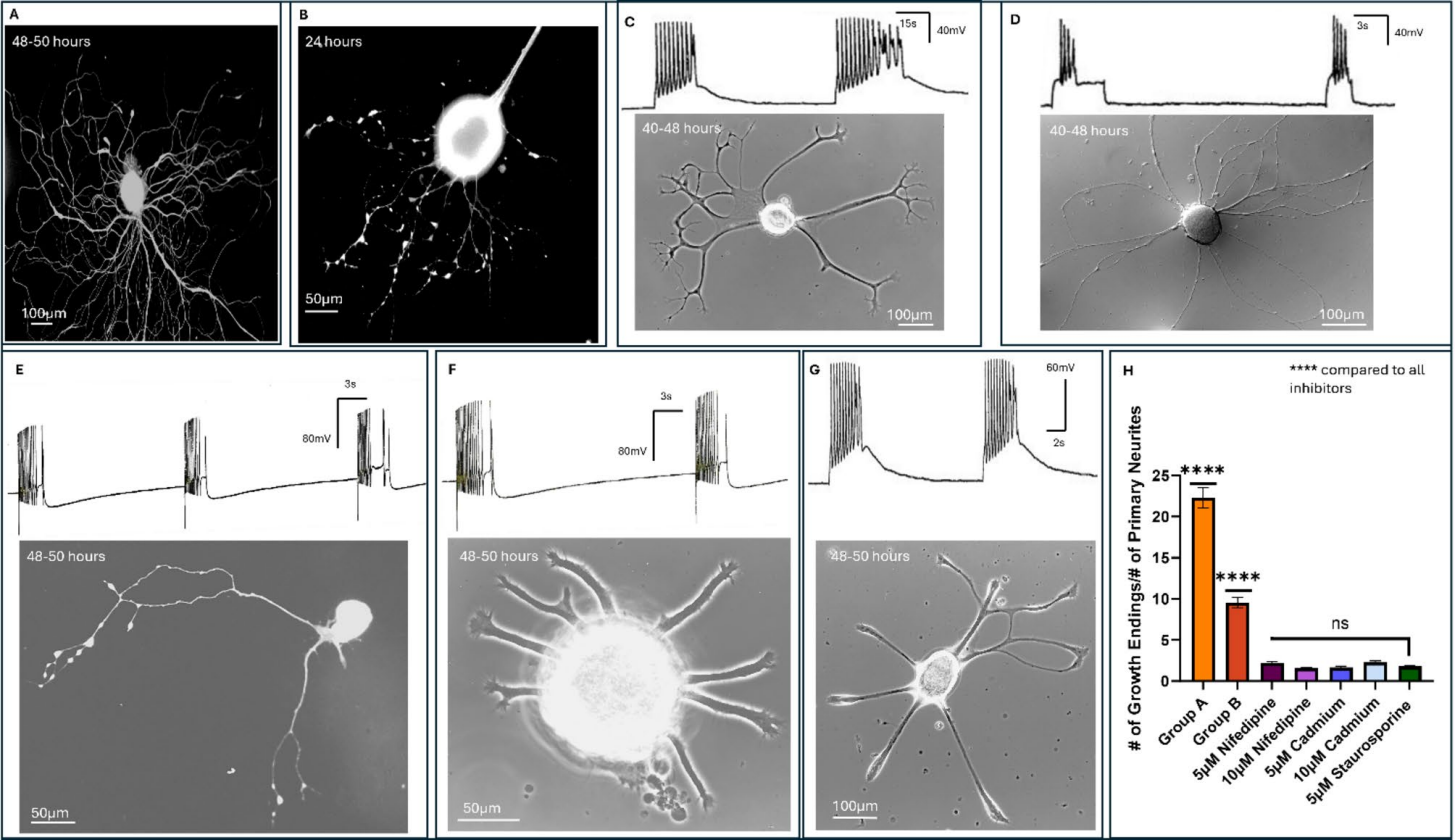
Activity evoked calcium influx regulates the extent of growth in LPeD1 neurons. (**A**) Representative non-ratiometric calcium imaging of a LPeD1 neuron during endogenous activity using Fluo-4 dye (at 48–50 h) (*n* = 7). **(B)** Non-ratiometric calcium imaging using Fluo-4 dye for LPeD1 growth cones at 24 h during exogenous bursting stimulation (*n* = 9). **(C)** LPeD1 neuronal growth (after 40–48 h) when the neurons were cultured in the presence of 5 μm (*n* = 16 added after 2–4 h of plating) and **(D)** 10 μm nifedipine (*n* = 11). **(E)** LPeD1 neuronal growth (photographed at 48–50 h) when cultured in the presence of 5 μm (*n* = 20), and **(F)** 10 μm cadmium (*n* = 25 added after 2–4 h of cell plating). **(G)** Blocking protein kinase A with 5 μm staurosporine (cells photographed after 48–50 h of cell plating) inhibited neuronal branching in LPeD1 neurons (*n* = 18, the drug was added after 2–4 h of cell plating). For all inhibition experiments, exogenous stimulations (as above) were elicited (0.1-0.2nA) in attempts to induce branching, while also monitoring the health of neurons. **(H)** Ratio of the number of growth endings to number of neurites present after 48-hours for unperturbed LPeD1 neurons in Groups A and B, as compared to instances of inhibiting Ca^2+^ or PKA. **Statistics: (H)** used a one-way ANOVA (F[6, 99] = 368.1, *P* < 0.0001) followed by Tukey’s multiple comparisons test to compare all Ca^2+^ inhibitors alongside staurosporine to Group A and B neurons, alongside staurosporine to all Ca^2+^ inhibitors. A Holm-Bonferroni correction was applied to all p-values to account for family wise error rate. Error bars indicated SEM. A scale bar is provided for all images.

Both spontaneous and induced activity invokes Ca^2+^ fluxes which are deemed important for cellular viability, growth, neuronal activity, and synaptic connectivity. Here we sought to determine whether both spontaneous and induced activity patterns would invoke intracellular Ca^2+^ fluxes, and if their perturbation impacted growth accordingly. LPeD1 neurons were cultured as above and allowed to exhibit growth. Non-ratiometric Fluo-4 dye was then added to the culture dishes after 12–18 h of neuronal sprouting. We observed that both spontaneous (Fig. 3A, *n* = 7) and induced activity (Fig. 3B, *n* = 9) in LPeD1 neurons raised intracellular Ca^2+^ throughout the cell in all instances. We also observed Ca^2+^ rises in the growth cones during both spontaneous and depolarized neurons. Given that the Fluo-4 dye used does not permit ratiometric quantification, the exact concentrations of intracellular Ca^2+^ could not however be deduced. Moreover, a potential pitfall regarding the use of ratiometric dyes, such as Fura-2, over an extended period was its compartmentalization in various cellular organelles and the ensuring cytotoxicity.

To further investigate the involvement of intracellular Ca^2+^ fluxes in growth patterns induced by electrical activity, we cultured LPeD1 neurons in the presence of either 5µM or 10µM nifedipine. Nifedipine is an L-type Ca^2+^ channel blocker, which we have shown previously to block intracellular Ca^2+^ influx from L-type channels in these neurons^28^. We observed that 5µM nifedipine perturbed growth but still allowed some branching to occur (Fig. 3C, *n* = 16). It was interesting to note that although neurons cultured in the presence of 10µM nifedipine exhibited some growth, they were generally devoid of branching, mimicking the instances where the cells were hyperpolarized via direct current injections **(**Fig. 3D, *n* = 11**)**. For neurons cultured in both concentrations of nifedipine, we stimulated the neurons using 0.1-0.2nA current injection to decipher their physiological health and found them to be electrophysiologically viable (resting membrane potential and spikes). Since nifedipine does not block all types of Ca^2+^ channels, we next used a non-specific Calcium channel blocker. Cadmium at concentrations of 5µM (Fig. 3E, *n* = 20) and 10µM (Fig. 3F, *n* = 25) blocked neurite outgrowth in a concentration dependent manner **(**Fig. 3E**)** in neurons that were otherwise electrophysiologically viable (resting membrane potentials and somewhat compromised spikes). Taken together, these data demonstrate that both induced and spontaneous activity patterns in LPeD1 neurons invoke Ca^2+^ fluxes which are required for normal growth in all tested cells.

Protein kinase A (PKA) has been implicated in guiding neuronal branching through phosphorylation of transcription factors such as CREB and microtubule-associated proteins (MAPs)^29^ downstream from Ca^2+^ signaling. We therefore asked the question whether PKA might also be involved in regulating the growth of LPeD1 neurons. Specifically, to determine whether these activity-dependent mechanisms regulating neuronal branching involved PKA, the unpaired LPeD1 were cultured in the presence of 5µM staurosporine, a PKA inhibitor. We observed that under these experimental conditions the cells failed to sprout even when stimulation was applied throughout **(**Fig. 3G, *n* = 18**)**. To determine if our time-lapse imaging might have missed finer or thinner neurites, the cells were fixed and labelled with anti-tubulin antibody and fluorescent images acquired and growth patterns assessed. In comparison to Group A and B neurons, we observed that all concentrations of nifedipine, cadmium, and staurosporine significantly decreased branching (quantified by ratio of growth endings to primary neurites) (*p* < 0.0001 for all comparisons) (Fig. 3H). However, after accounting for multiple comparisons, we did not observe a significant difference in the number of neurites between staurosporine, and any of the concentrations of nifedipine or cadmium (*p* > 0.05 for all comparisons). Altogether, these data validate previous findings that Ca^2+^ fluxes are required for neuronal growth to occur, and that the absence of intracellular calcium influx inhibits branching from occurring through a downstream pathway that likely involves a PKA mechanism.

## Microelectrode array recordings reveal how neuritic contact alters activity patterns

The above neuronal interrogations made with direct intracellular recordings; while providing in-depth analysis of an association between neuronal activity and cellular growth patterns, they revealed only a snapshot which was restricted to either a limited time window or fewer neurons that could be monitored concurrently. Specifically, the sharp electrode recordings did not permit monitoring of the activity in the early hours following plating, or at later (10–20 h) time windows due mainly to the experimental limitations. Specifically, neurons required firm adhesion to the dishes and if impaled sooner, they got stuck to the electrodes and detached from the substrate, whereas longer than 6-hour continuous intracellular recordings had the potential to compromise cellular viability and health. Therefore, to determine the relationship between activity and growth, both immediately after plating and beyond a 6–8-hour time window (up to 60 h), we leveraged microelectrode arrays (MEAs) to enable long term recordings.

LPeD1 was either plated by itself (unpaired condition, *n* = 7), plated with its presynaptic partner VD4 at a short distance (growth-dependent synapse formation: GDSF condition, *n* = 8), or in a soma-soma configuration with its presynaptic partner (cell bodies juxtaposed against each other to facilitate synapse formation in the absence of growth^30^, *n* = 8) but in the presence of CM (a requirement for synapse formation).

**Fig. 4:**
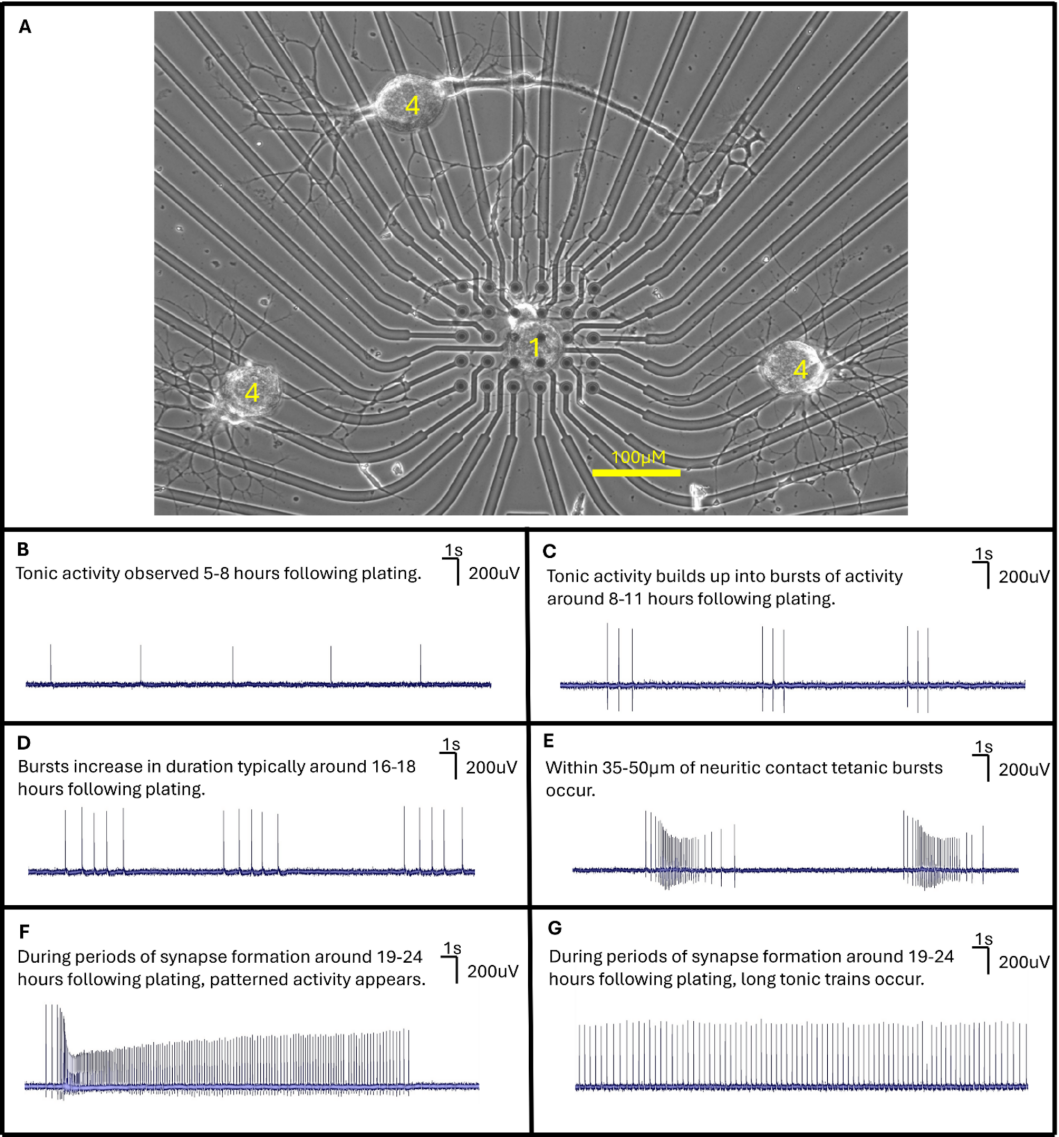
Characteristic activity patterns of LPeD1 neurons plated with VD4 on MEAs. **(A)** Example of individual LPeD1 and VD4 neurons plated on MEA exhibiting growth. 1 refers to LPeD1, 4 refers to VD4, a relative scale bar is provided for the image. **(B)** LPeD1 neurons predominantly exhibited either single spikes that were interspaced with prolonged periods of quiescence or prolonged tonic activity during the first 5–8 h of following plating (*n* = 8). **(C)** Spontaneous bursting activity began occurring consistently after 8–11 h following plating (*n* = 8). **(E)** These bursts began to consist of more spikes nearing the 16–18 h of recording (*n* = 8). **(F)** Tetanic activity was often seen during or following physical contact between the synapsing pairs (generally when growth cones were within 35–50 μm of target, *n* = 6 clear instances of contact between partners). **(G)** Nearing synapse formation which usually occurred around 19–24 h, long tonic trains would also occur (*n* = 6).

We first sought to determine if the activity patterns observed on MEAs were similar to those seen during sharp electrode recordings **(**Fig. 4A**)**. After letting neurons recover from extraction for four hours following plating, we observed distinct activity patterns **(**Fig. 4B-G**)** in the subsequent 30 h as neurons underwent growth and synapse formation. Both pre- and postsynaptic neurons exhibited neuronal branching and growth throughout recordings, which suggested that the MEAs neither compromised cellular viability, nor neuronal activity **(**Fig. 4A**)**. Specifically, we observed tonic spiking in the early hours of activity, typically 5–8 h post-plating **(**Fig. 4B**)**. Around 8–11 h into recordings, we observed tonic spikes begin to cluster into pairs of spikes which transitioned into consistent bursting activity **(**Fig. 4C**)**. For paired LPeD1 neurons, branching generally began to occur after 10 h post-plating. These bursts would exhibit increased number of spikes per burst around generally 16–18 h after plating (Fig. 4D). We observed subsets of tonic and bursting activity in a manner like that of the intracellular recordings. This included what we defined as tetanic activity when the growth cones of GDSF neurons were on average within 35–50 μm of physical contact with their presynaptic partner **(**Fig. 4E**)**. During tetanic activity, LPeD1 neurons would fire a burst whereby the last few spikes were of lower amplitude. During periods of synapse formation (typically around 19–24 h following plating), we would observe alternating patterns of bursting and tonic activity (Fig. 4F). During the periods of tetanic activity, we also observed long tonic spike trains (Fig. 4G). These observations were made in 8 LPeD1 neurons cultured at a distance from VD4, with 6 instances of LPeD1 forming clear neuritic contact with VD4. The above data suggested that neurons recapitulated the same activity patterns when cultured on MEAs as observed during sharp electrode recordings.

**Fig. 5:**
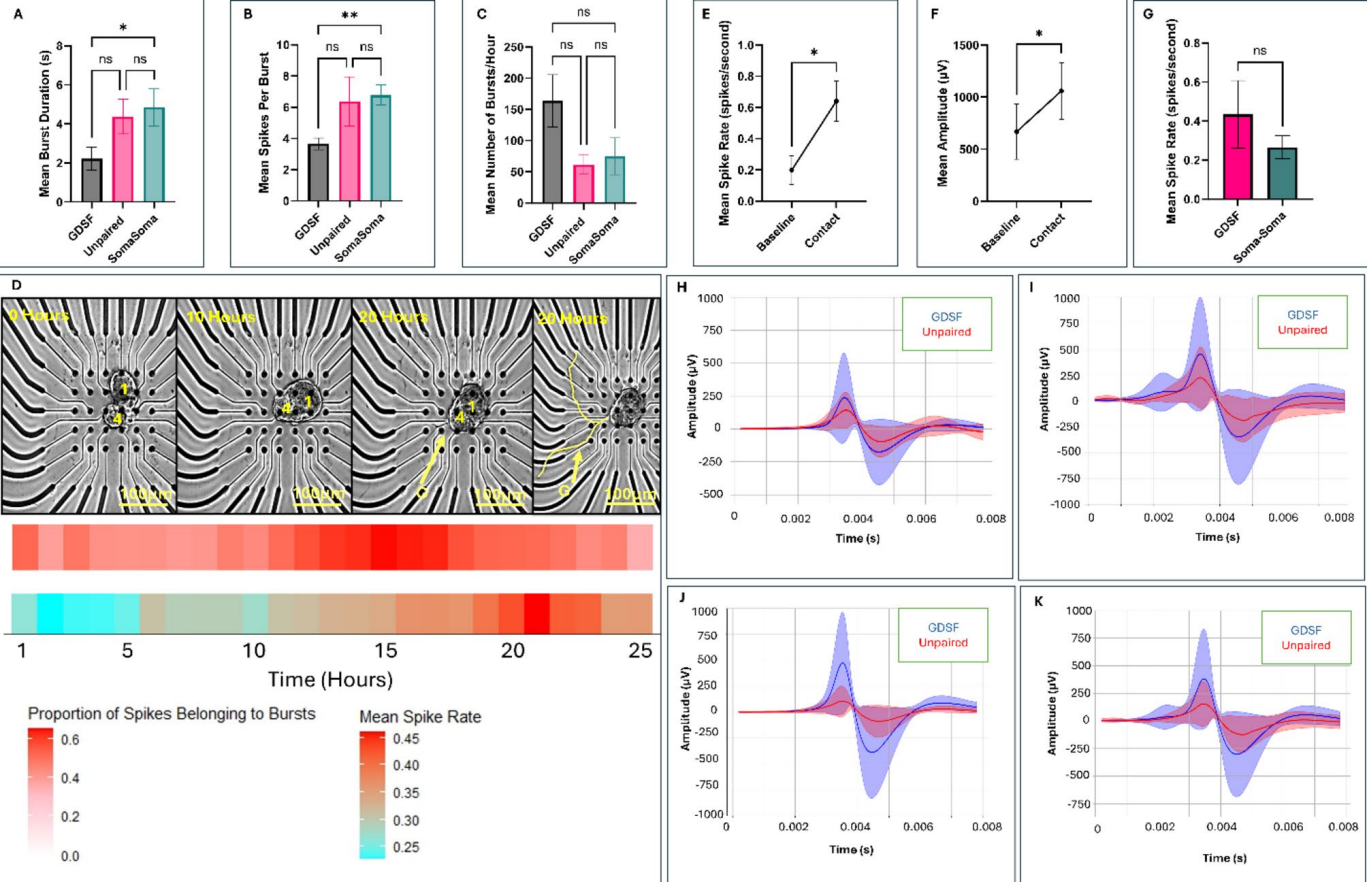
Presence and the pairing configuration of a synaptic partner modulates activity patterns. (**A**) Burst duration (seconds), **(B)** number of spikes per burst, and **(C)** number of total bursts recorded from postsynaptic LPeD1 neurons under different culture configurations during the 10 h post plating (GDSF: *n* = 8, Unpaired: *n* = 7, Soma-Soma: *n* = 8). **(D)** Representative timelapse and corresponding raster plots for neurons cultured adjacently to each other (*n* = 8). ‘G’ indicates when growth began, 1 refers to LPeD1, and 4 refers to VD4. Burst heatmap is provided directly underneath timelapse where the proportion of spikes belonging to bursts within an hour bin uses a color gradient (low = white, high = red). Mean hourly spike rate is presented in a heatmap with a color gradient (low = blue, high = red). **(E)** Change in amplitude (µV) and **(F)** spike rate (spikes/s) from start of experiment to point of contact for LPeD1 neurons cultured separated from presynaptic VD4 (*n* = 6 neurons that made clear contact and network maturation). **(G)** Spike rate in neurons that branched to form synapses (*n* = 6) as compared to neurons cultured adjacently to each other to form synapses in the absence of growth (*n* = 8). **(H)** Comparison of spike waveforms between GDSF (*n* = 8) and unpaired (*n* = 7) conditions during hours 0–5, **(I)** hours 5–10, and **(J)** hours 10–15 of experiment, and **(K)** for hours 0–15 following recovery from plating. The average GDSF waveform is indicated with a blue line, the shaded blue region indicates SD. The average unpaired waveform is indicated with a red line, the shaded red region indicates the SD. Statistics: **(A)**, **(B)** and **(C)** used a Kruskal Walis Test (Kruskal-Walis Statistic: 7.800 (*P* = 0.0202), 9.026 (*P* = 0.0110), 4.005 (*P* = 0.1350) for **(A)**, **(B)**, and **(C)** respectively) with Tukey’s Honestly Significant Difference Test. **(E)** and **(F)** used a Wilcoxon paired test. **(G)** used a Mann-U-Whitney Test. **(H)**,** (I)**,** (J)**, and **(K)** used the Pearson Correlation Coefficient. Error bars indicate SEM. n.s *p* > 0.05, *p < 0.05, *p < 0.01.

Having established our two modes of recordings, we then sought to characterize how activity patterns change in the hours following cell plating and recovery from enzymatic extraction. During the first 10 h of post-plating, we observed that while neuronal activity was present throughout, neurons generally did not undergo significant growth or neuritic branching. This differed as compared to paired neurons used for intracellular recordings which sprouted around 6–8 h, suggesting that the electrode interface (potentially through static charge), delayed the initiation of neuronal growth or that the neuronal adhesion to the MEA substrate took longer than direct attachment to the polyline. When neurons were paired in a soma-soma configuration (*n* = 8), we observed significantly longer bursts in LPeD1 as compared to LPeD1 neurons that were physically separated (plated at a distance, *n* = 8) from their synaptic partner (Fig. 5A, *p* = 0.02961). These bursts also consisted of more spikes (Fig. 5B, *p* = 0.0085) however, the number of hourly bursts did not differ significantly between different culture conditions (Fig. 5C, *p* = 0.1350). LPeD1 neurons cultured in soma-soma configurations did not however demonstrate branching until 15–20 h into recordings (Fig. 5D, *n* = 8). We then sought to determine how neuronal activity changed leading up to neuritic contact between cells that were plated at a distance from each other. Our findings demonstrated an increase in spike rate (*p* = 0.0313) and amplitude (*p* = 0.0313) from baseline firing (defined as first hour of recorded neuronal activity) to the point of contact (Fig. 5E-F, *n* = 6). We then sought to determine if there were differences in spike rates following synapse formation in GDSF conditions (*n* = 6, where clear contact and network maturation occurred between synaptic partners) and soma-soma paired neurons (*n* = 8). That is, to determine if the cell-cell contact from the start altered neuronal activity. We found no statistically significant differences in spike rates between GDSF and soma-soma neurons (Fig. 5G, *p* = 0.6620).

We next asked whether the spike waveforms themselves differed in neurons when they were cultured in the presence of a synaptic partner at various stages of development. The Pearson Correlation Coefficient (PCC) was used to determine if their waveforms exhibited any similarities. We found that at the start (first 5 h) of the experiment the PCC between unpaired and GDSF waveforms was 0.9640 (*p* = 0.0005). In the middle (hours 5–10) of the experiment, it was 0.9529 (*p* = 0.0009), and towards the end (hours 10–15) it was 0.9558 (*p* = 0.0008) **(**Fig. 5H-J**)**. The overall PCC between unpaired and GDSF waveforms was 0.9644 (*p* = 0.0005, Fig. 5K). Altogether, this suggests a near perfect linear correlation between GDSF and unpaired spike waveforms, irrespective of the various growth phases of the neurons. The above data suggested that while activity patterns may change at different stages of development, the waveforms themselves remain largely unchanged.

### Burst frequency continued to decrease in the hours following synapse formation

**Fig. 6:**
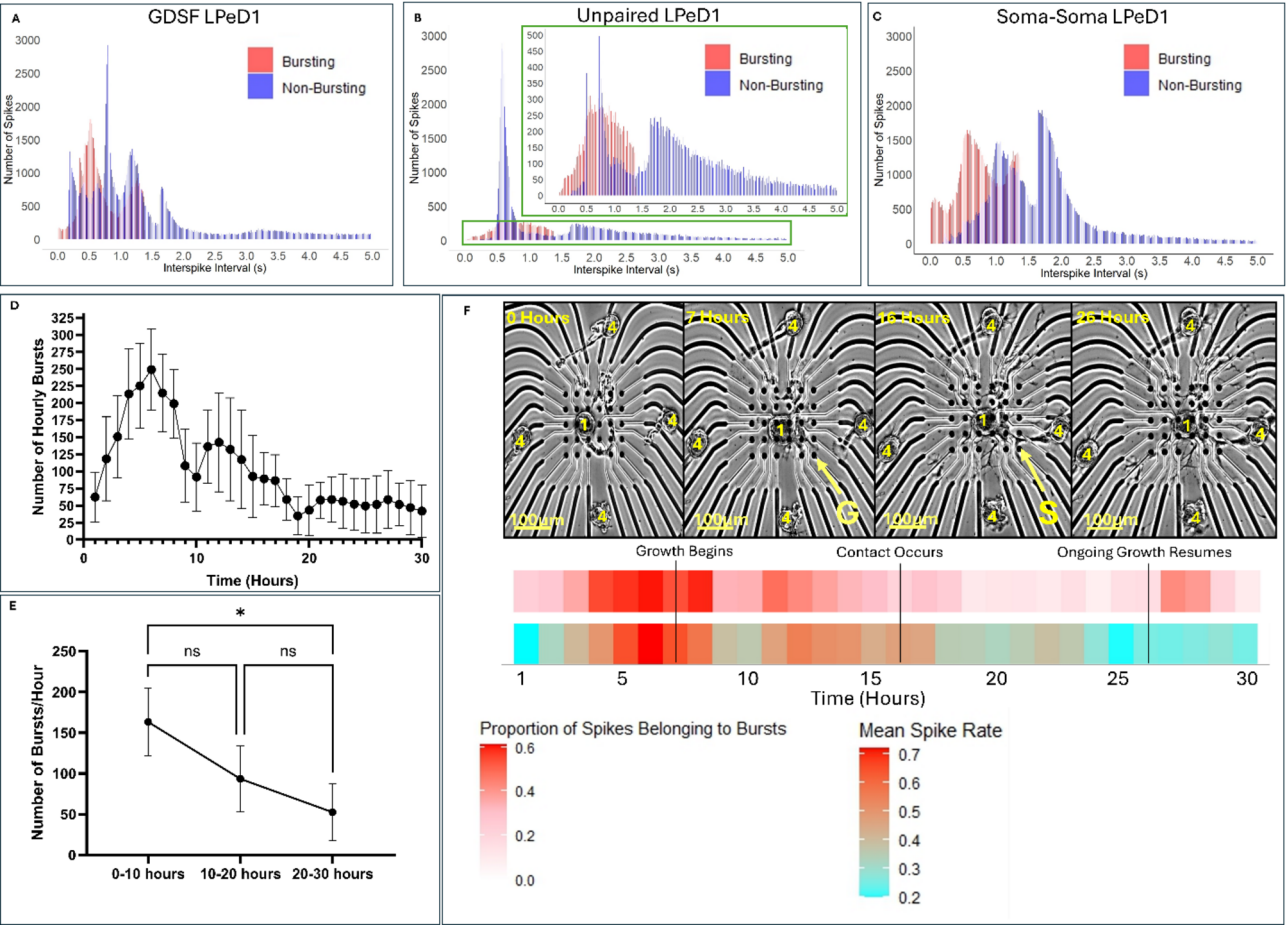
MEA recordings validate role of bursting activity as observed in intracellular experiments. (**A**) Distribution of all burst patterns recorded from LPeD1 neurons that were cultured separated from their synaptic partner VD4 (*n* = 8). **(B)** Distribution of all bursts recorded from unpaired LPeD1 neurons. Zoom in provides focus on the main part of the distribution (*n* = 7). **(C)** Distribution of all bursts recorded from LPeD1 neurons when cultured adjacently with their synaptic partner VD4 in a soma-soma configuration (*n* = 8). For histograms bursts are indicated in red, non-bursting activity is in blue. **(D)** Number of hourly bursts recorded from LPeD1 neurons cultured separated from their synaptic partner VD4 throughout 30 h of development following plating (*n* = 8). **(E)** Mean hourly bursts recorded from LPeD1 cultured separated from VD4 throughout 10-hour intervals (*n* = 8). **(F)** Representative timelapse of postsynaptic LPeD1 establishing physical contact with its presynaptic partner VD4 (*n* = 6) with mean raster plots of corresponding activity patterns. Burst raster plot is provided directly underneath timelapse where the proportion of spikes belonging to bursts within an hour bin uses a color gradient (low = white, high = red). The overall mean hourly spike rate is presented in a heatmap with a color gradient where blue indicates the lowest spike rate in a one-hour time bin during the experiment, and red represents the highest spike rate in a one-hour time bin. ‘G’ indicates when growth began, S refers to synapse formation, 1 refers to LPeD1, and 4 refers to VD4. A scale bar is provided for all images. Image quality was sharpened to improve visibility of growth when scaling for figure panels. Statistics: **(E)** used a Friedman test (Friedman statistic: 7.000, *P* = 0.0303) with Tukey’s Honestly Significant Difference Test. Error bars indicate SEM. n.s *p* > 0.05, *p < 0.05.

Having characterized the activity in the early hours of neuronal culture, we then sought to determine how this activity changed over time following synapse formation. We classified bursting activity based on interspike intervals (ISI) to determine burst spike distributions relative to non-bursting spikes for the three culturing configurations **(**Fig. 6A-C**)**. In analyzing these distributions, the overlap with some tonic spikes is likely indicative of the long tonic spike trains described previously. We observed that bursting frequency decreased leading up to the 30-hour mark in neurons that were cultured at a distance from their synaptic partner **(**Fig. 6D**)**. As predicted from our sharp electrode recordings, there was a statistically significant decrease in the number of bursts in these neurons following 20 h of recordings (Fig. 6E, *p* = 0.0373, *n* = 8). We observed an increase in bursting frequency which corresponded to the initiation of neuritic branching **(**Fig. 6F**)**. Upon synapse formation (based on our previous findings where synapses were shown to form 50–60 min following contact between *Lymnaea* synaptic partners^20^, bursting activity however, decreased in frequency as observed during our sharp electrode recordings. This decrease in bursting frequency was particularly interesting given that the spike rates at times increased following synapse formation, but this correlated with a transition towards tonic activity as seen during the early hours following plating. This observation corroborated with the intracellular recordings, and further demonstrates the importance of bursting activity in neuritic branching and synapse formation.

## Discussion

All excitable cells exhibit unique attributes that distinguish them from other non-excitable cells. It is important to note that despite the evolutionary divergence among species, fundamental physiological processes in all excitable cell types ranging from resting membrane potential, action potentials, impulse conduction, to outgrowth, synapse formation, synaptic plasticity etc. remain largely conserved. These attributes also include transmitter secretion capabilities and neurotransmitter receptor functions in neurons^31^. Consistent with this notion are previous studies that have demonstrated that morphologically and functionally homologous neurons from different species can form inter-species synapses in vitro^32^, suggesting that the fundamental mechanisms underlying neuronal excitability and synapse formation are also evolutionarily conserved across diverse species^32^. Thus, the insights obtained here regarding interdependence of electrical activity, neuronal growth patterns and synapse formation could justifiably be extended to other animal species as well.

One of most outstanding question in the field of neuroscience is how various neuron types acquire their distinct patterns of polarity, and what are the underlying triggers that help them achieve their respective morphologies. Here we identified electrical activity patterns as being one of the factors that regulated distinct patterns of growth in the LPeD1 neuron. Specifically, we provided direct evidence that unique patterns of electrical activity help shape distinct branching in LPeD1 which involved Ca^2+^ influx and PKA pathway. These data align with studies in vertebrates where neuronal depolarization of the rat supraoptic nucleus has been shown to induce increased dendrite branching^33^. Similar activity mediated mechanisms have also been described in primary hippocampal cultures of neonatal Swiss mice pups where axonal growth cones were stalled following their electrical stimulation^34^. The authors proposed that growth cone motility is controlled by transient neuronal bursts, consistent with what we demonstrated here. Additionally, we demonstrated here that hyperpolarizing LPeD1 neurons although reduced axonal branching but it did not perturb neurite outgrowth, in a manner analogous to that of vertebrate models^35^. Similarly, blocking neuronal activity has been shown to decrease axonal branching within the neonatal mouse visual cortex^36^.

Whereas the role of Ca^2+^ fluxes in neurite outgrowth has been well established, less is known regarding the downstream pathways. PKA has been proposed to play a key role in guiding neurite extension and branching when activated through adenylyl cyclase (cAMP) binding to its regulatory subunits, which in turn causes increased phosphorylation of CREB, and subsequent transcription of growth factors^37^. Neuronal activity and the resulting intracellular Ca^2+^ fluxes can increase the levels of cAMP thus phosphorylating a host of proteins involved in various cellular functions. Targeted inhibition of PKA in mouse hippocampal neurons has been shown to result in reduced neurite elongation^38^. However, there appears to be a dose-dependent effect where both hyperactivation or inhibition of PKA can result in decreased neuritic outgrowth^39^. In *Drosophila*, perturbations of PKA signaling has been shown to limit dendrite length as well as the number of branches^40^. In this study, we have demonstrated that inhibiting PKA decreased neuritic extension and branching even when neurons were induced to exhibit electrical activity. Our findings where we inhibited intracellular Ca^2+^ fluxes using nifedipine and cadmium point towards Ca^2+^ signaling which may have resulted in the activation of PKA leading to neuritic branching. To demonstrate the role of PKA unequivocally in regulating neurite outgrowth further experiments would however be required.

While using defined synaptic partners VD4 and LPeD1 enabled interrogation of these fundamental activity mediated mechanisms, it is important to acknowledge the absence of glial influences when comparing our data to vertebrate models. Specifically, Hughes et al. demonstrated that GABAergic hippocampal neurons cultured with astrocytes had increased complexity in their axonal arbors as compared to cells cultured without astrocytes^41^. The authors demonstrated that astrocytic proteins could modulate neurite outgrowth thus underscoring the importance of glia in the regulation of specific growth patterns. Additionally, Zhu et al. found that astrocytic release of phospholipase D1 was able to regulate dendritic branching and extension in neurons^42^. Taken together with our data, these previous findings in both vertebrate and invertebrate models suggest that while neuronal activity mediated mechanisms can induce morphological changes such as neuritic branching, astrocytes are also able to modulate these processes further. It is however important to note that *Lymanea* neurons do not require glia for their growth and synapse formation as in vivo these non-neuronal cells are present only in the neuropile area and not around the somata or the axons.

Regarding the importance of electrical activity in development, electrical stimulation has been shown to even affect neural stem cell fate and function in vitro^43^. Moreover, brief electrical stimulation of cultured Xenopus spinal neurons was shown to elicit both positive and negative growth cone turning responses towards growth permissive and suppressive molecules respectively^44^. Since vertebrate dendrites are the site of most synaptic contacts, thus the extent of their branching would likely determine the number of synaptic contacts that a neuron might receive^44-46^. In addition to growth permissive role of electrical activity, the spontaneous activity has also been shown to regulate dendritic pruning^47^. Extensive dendritic pruning, is thought to leads to severe neurodevelopmental disorders such as intellectual disabilities, demonstrating the importance of appropriate dendritic branching in proper wiring of the brain^48,49^. Our results showed that increased number of spikes within spontaneous bursting activity did indeed exhibit more elaborate branching, but we do not know if these neurons would be primed for greater synaptic partnership. A direct comparison with the vertebrate models would however be difficult to make as the invertebrate neurons do not exhibit a clear demarcation between dendrites and axons due to differences in their cellular polarity. However, it seems safe to infer that subtle differences in bursting activity may alter the extent of neuritic branching, and that the bursting activity may serve as a trigger for branching; this had not been shown previously.

The evidence suggests that perturbing activity during early development prevents proper network development^50,51^. However, this has often been achieved by irreversibly inhibiting either genes underlying voltage-gated channels, or through toxins^50,51^. In both instances, the approach led to irreversible changes in neuronal physiology. Here we demonstrated for the first time that physiological perturbation of electrical activity by mere membrane potential manipulation served to alter neuronal growth patterns in a reversible manner. Moreover, we demonstrated that within minutes of contact between the growth cones, synapses are formed and that this synaptogenesis further changes the firing patterns of neurons underscoring a direct relationship between electrical activity and synapse formation.

Previous research has demonstrated that blocking intracellular Ca^2+^ using nifedipine and cadmium inhibits dendrite extension and synapse formation^52^. However, there is also evidence demonstrating that decreasing intracellular Ca^2+^ levels accelerates neuritic extension^53^. The challenge in consolidating these findings on the role of Ca^2+^ in guiding neuritic extension arises from the multitude of Ca^2+^ channel subtypes. This can make it difficult to decipher the precise role of any specific Ca^2+^ channels and their ascribed function in regulating neuritic branching and neuronal morphology. However, in our single cell model system, these challenges could be mitigated. We have previously shown that nifedipine blocks L-type Ca^2+^ channels but does not completely abolish intracellular Ca^2+^ influx from other voltage gated channels^54^. The addition of nifedipine resulted in growth patterns similar to those when neurons were hyperpolarized and both perturbations inhibited branching. This supports the notion that activity dependent mechanisms guide growth and branching. Cadmium, though being a non-selective Ca^2+^ channel blocker, reduced Ca^2+^ to a level that arrested growth initiation and may have also compromised neuronal health.

We have previously shown that soma-soma cell contact increases intracellular Ca^2+^ levels which in turn inhibited neurite outgrowth in these paired neurons^30^. Our previous work highlighted that neurite suppression from soma-soma pairs occurs with a delay of 48–72 h, which is corroborated by our findings in this study.

With the advent of MEA technology, there is a desire to switch away from conventional intracellular and path-clamp recordings. We put this notion to test in this study and while using MEAs, we were able to monitor longer bursts over an extended period from multiple neurons, this approach was however devoid of the resolution that the intracellular recordings rendered. It is therefore important that until such time that when the resolution of MEA recordings become significantly higher, the conventional sharp electrode approach would remain desirable for these experiments as it allows for both the stimulation and silencing of neurons within a physiological range.

Target cell contact has previously been shown to suppress neurite outgrowth following synapse formation in leech neurons as well^55^. Contact made by growth cone filopodia has been shown to change its direction towards the target cell followed by stabilizing contact with the synaptic partner^56^. This suggests that following cell contact and synapse formation, morphological changes occur throughout neurons as they negotiate their long-term synaptic partnership. In this study, we observed a decrease in burst frequency following synapse formation suggesting that during and following synapse consolidation, neuronal activity patterns undergo a transformative change. Previous studies have shown that cell-cell contacts between synaptic partners may bring about specific morphological and molecular changes in their respective partner cells^57-59^, which would likely involve mechanisms other than the electrical activity. Since synaptogenesis is proceeded by dendritic or axon growth, it is therefore safe to assume that intrinsic electrical activity may be the key player in defining both the growth and branching of developing neurons.

Combining sharp electrode and MEA techniques for electrophysiology analysis provided several advantages. Sharp electrodes allow for higher resolution intracellular recordings^60^, which was particularly valuable during key developmental stages such as branching and synapse formation. This difference in resolution was particularly evident with our MEA recordings capturing voltages in the microvolt range, whereas sharp electrode recordings consistently captured voltages in the millivolt range. Sharp electrode recordings do however have limitations, such as potential leak of the recording solution between the electrode and the cell body^61^. Additionally, sharp electrodes are an invasive process^62^, whereas MEAs enabled long-term characterization of activity from plating to the hours following synapse formation. This longitudinal data revealed trends in neuronal activity following the maturation of synaptic connections. Additionally, through MEAs, we were able to deduce the activity patterns present in the hours prior to the sharp electrode recordings. During those periods, we observed prolonged instances of tonic activity which eventually built up into bursts, leading up to neuronal growth initiation. Our observation of different types of activity patterns in the hours prior to when sharp electrode recordings were performed suggests that early neuronal activity prior to growth initiation was perhaps meant to prime the neurons for the ensuing growth.

In conclusion, here we explored the role of activity-mediated mechanisms underlying growth and synapse formation. By leveraging both sharp electrode and MEA recordings, we characterized the role of activity during both specific growth stages of a neuron, and throughout its growth. Taken together, this study sheds light on how disruptions of electrical networks can reversibly halt or impair synaptic formation in this model system. These findings further suggest the possibility of manipulating activity-mediated mechanisms, either directly or pharmacologically to correct abhorrent growth or miswiring in a developing network.

## Methods

### Animals

Freshwater snail *Lymnaea stagnalis* were used in all experiments. Animals were fed romaine lettuce twice a week and kept in a well-aerated aquarium at room temperature (20–22 °C) with 12 h day-night cycle. The central ring ganglia were isolated, and neurons cultured in brain conditioned media (CM) as described previously^63^. Individually identified and functionally characterized pre- and postsynaptic neurons Visceral Dorsal 4 (VD4) and Left Pedal Dorsal 1 (LPeD1) respectively were cultured either as single cells or in pairs. The animals used for preparing CM were between 2 and 6 weeks old, whereas neurons were isolated from animals that were 1–2 months old as described previously^64^.

### Dissections

Animals were placed in *Lymnaea* saline solution (in mM: 51.3 NaCl, 1.7 KCl, 4.0 CaCl2, and 1.5 MgCl2 buffered to pH 7.9 with HEPES). Following de-shelling with forceps, they were placed in a 10% Listerine solution (ethanol, 21.9%; methanol, 0.042%) for 10 min to anesthetize the snails. The animals were then pinned down on a Sylgard dish containing *Lymnaea* saline containing antibiotic (50 µg/L gentamicin). The central ring ganglia were then treated with trypsin (2 mg/ml) for 18 min and then with trypsin inhibitor (2 mg/ml) for 10 min as described previously^63,65^.

### Cell culture

*C*entral ring ganglia were isolated and washed 6 times using *Lymnaea* saline with antibiotic (50 µg/L gentamicin), each wash was approximately 15 min. CM was made using 12 *Lymnaea* brains which were incubated in 6mL of defined media (DM), consisting of DM-serum-free 50% L-15 medium with 20 µg/L gentamicin and inorganic salts at mM: 40 NaCl, 1.7 KCl, 4.1 CaCl2, 8 and 1.5 MgCl2 and 10 HEPES at pH 7.9. The brains were removed post 3 days incubation at room temperature, and this medium was termed CM-1. The same brains were then used again as above, after several antibiotic washes but were instead incubated in DM for 4 days, the isolated solution being called CM-2. This was repeated one last time after incubation for 6 days, producing CM-3 which was stored at -20 °C. CM-3 contains the greatest concentration of trophic factors and was used for experiments in accordance with our previous experiments in this model system^66^. LPeD1 and VD4 were isolated from 1 to 2 months old *Lymnaea* using the protocol as outlined^67^. Briefly, identified LPeD1 and VD4 neurons were isolated by applying gentle suction through a fire-polished, Sigmacote (Sigma, St. Louis, MO)-treated pipette. This pipette was attached to a micro syringe filled with high osmolarity DM (D-glucose, 20mM), attached to a micromanipulator. These individual neurons were cultured onto the poly-l-lysine coated MEA. Neurons were plated in CM onto the MEA before moving to recording set-up. An isolated LPeD1 neuron was either cultured onto an MEA by itself as a control, with the presynaptic partner VD4 either separated in the growth dependent synapse formation (GDSF) condition, or in a soma-soma configuration where the two neurons were directly adjacent to each other.

Falcon 3001 culture dishes were cleaned and sterilized at least 48 h prior to the day of experiment. Within 24 h of the experiment day, 200µL of poly-L-Lysine (1:1 solution of poly-L-Lysine in Tris buffer pH 8) was added to the glass cover slip glued dishes as described previously^66^. LPeD1 and VD4 were isolated and cultured in the same manner described for MEAs, also in the presence of CM.

### Preparing meas

Microelectrode arrays (MEAs) were cleaned and sterilized at least 48 h prior to the day of experiment. Within 24 h before the experiment day, 1mL of poly-L-Lysine (1:1 solution of poly-L-Lysine in Tris buffer pH 8) was added to the MEA well as described previously^66^. These individual neurons were allowed to settle on the poly-L-lysine coated MEA. Neurons were grown in CM, and then left on MEA for 4 h to ensure proper adhesion before moving to recording system (Multichannel Systems MEA1060-Up-BC). Isolated LPeD1 were either cultured onto an MEA by itself as controls, or with its presynaptic partner VD4. Paired neurons were either cultured separated to allow for growth and the ensuing synapse formation between the neurites, or in a soma-soma configuration where the two neurons were juxtaposed against each other.

### Electrophysiology

#### Intracellular recordings

Conventional intracellular recording techniques were used as described previously^68,69^. Glass microelectrodes (1.5 μm internal diameter; World Precision Instruments, Sarasota, FL) were prepared with an electrode puller (Narishige PP-830, Tritech Research) and then filled with a saturated solution of K_2_SO_4_ yielding resistance of 20–40 Mega ohms. Neurons were viewed under an inverted microscope (Axiovert 135; Zeiss, Thornwood, NY) and impaled using Narashige (Tokyo, Japan) micromanipulators (MM 202 and MM 204). The electrical signals were amplified (Neuro Data Instrument Corp.) and displayed on a digital storage oscilloscope (PM 3394; Philips, Eindhoven, The Netherlands) and recorded on a chart recorder (TA 240 S; Gould, Cleveland, OH). For recording endogenous neuronal activity, sharp electrodes were inserted 6–8 h after plating, as neurons began to sprout. We recorded for 8–12 h following neuronal sprouting, which was the period during which the most robust growth occurred. This included making recordings at 12-, 18-24-, and 48–50 h post plating. Synapse formation was verified using concurrent intracellular recordings from LPeD1 and VD4 neurons to verify the presence of excitatory post-synaptic potentials in LPeD1. Neither depolarizing or hyperpolarizing currents were injected directly via the amplifier or using a Grass Stimulator (Astro-Med Grass Instrument S48 Stimulator Lab). For hyperpolarization experiments, a sharp electrode was inserted 4–6 h after plating, prior to neurons exhibiting growth. The sharp electrode remained in place delivering hyperpolarizing current into the neurons until 48–50 h post-plating. Concurrent time-lapse recordings were made using Panasonic video recorder and cells also photographed with a Nikon camera equipped with intervalometer (30 s. to 1 min interval).

### Microelectrode array recording

Cells plated on MEA were mounted on a MultiChannel Systems commercial MEA amplifier and PCI acquisition card. The amplifier was placed under a Zeiss Axio Observer Z1 microscope (Zeiss Crop., Ottawa, ON, Canada) used for recording and imaging. High density microelectrode arrays were used. MC_Rack software was used for displaying the data, and the sampling rate was set to 5 kHz for data acquisition.

### Imaging neurons during electrophysiology

A incubator-mounted Zeiss Axio Observer Z1 microscope (Zeiss Crop., Ottawa, ON, Canada) with a 5X objective was used for time lapse recordings of neurons on MEAs for up to 60 h. The microscope was used in a temperature-controlled imaging room, with built-in incubator which allowed the neurons to remain stable during long term recordings through temperature and humidity regulation controls. All recordings were conducted at 21˚C and 90% humidity. Imaging for neurons for sharp electrode electrophysiology was completed using an inverted microscope (Axiovert 135, Zeiss, Thornwood, NY) in the same temperature-controlled room. TeckPan film was used to photograph all neurons during sharp electrode experiments.

### Qualitative calcium imaging

Fluo-4 (1–4 μm) conjugated with Oregon Green was dissolved in DMSO (0.1%) and added to dishes containing the neurons for 10–15 min while acquiring intracellular recordings. Ca^2+^ fluctuations were monitored throughout the experiments during both spontaneous activity and when neurons were stimulated via direct intracellular stimulation. The dye was excited at 488 nm with an emission above 520 nm using an argon lamp. Non-ratiometric calcium imaging was used as ratiometric imaging can cause extensive Ca^2+^ intake into organelles, which can compromise the long-term recordings performed in this study.

### Inhibiting calcium channels and protein kinase A

Either nifedipine (5 μm and 10 μm) or Cadmium (5 μm and 10 μm) was added to culture dishes at the start of the experiment and neurons recorded/ monitored for 10–12 h. Protein kinase A was inhibited using staurosporine (MCE MedChemExpress) using a concentration of 5µM, as at higher concentrations staurosphorine is less selective in inhibiting protein kinase A.

### Immunostaining

Following 48–50 h post plating in the presence of calcium blockers or staurosporine, cultured LPeD1 were fixed for 30 min with 4% paraformaldehyde as previously described^70^. Anti-Tubulin mouse monoclonal antibody (obtained from Boehringer-Mannheim) alongside secondary antibodies (Vector Labs Inc.) was used to stain the neurons as described previously^70^.

### Counting neurites

The number of primary neurites and number of neurite tips (growth endings) was counted manually from LPeD1 neurons following 48 h post-plating. A ratio of the number of growth endings to the number of primary neurites was used to approximate the extent of branching. Neurons were considered sprouted when they had exhibited outgrowth equivalent to five soma diameter as previously described^26,27^. For experiments involving stimulation of LPeD1 neurons to assess changes in growth patterns, all cells used had already sprouted as defined by our criteria of neurites being longer than 5 soma diameters. We selected only those larger neurites that were in an active growth phase and distant from neighboring sprouts. We then focused only on those branches following either the release of repolarization, to allow resumption of endogenous activity or triggering depolarization via direct current injection through the intracellular electrode. This approach was adopted to ensure that only those branches under observation were monitored. The purpose of this experiment was to deduce whether the electrical activity manipulation would regulate the branching of individual neurites in a manner analogous to that of the global growth patterns.

#### Data processing

Spike train data from MEAs was obtained from MC_Rack during recordings, and the raw data was passed through a high pass filter with a threshold of 200 Hz. The filtered data was stabilized to the reference ground electrode. Spikes were characterized by a threshold of 10uV higher than the baseline (typically a threshold set around 40µV), a value determined through manual inspection to ensure accurate spike classification.

### Waveform analysis

The waveforms of 300 action potentials were saved for each neuronal MEA recording at the start, middle, and the end of each experiment. The action potentials were used to determine the average waveform of GDSF and unpaired neurons at different phases of development. The standard deviation (SD) of these averaged waveforms was determined and then the Pearson correlation coefficient (PCC) was calculated between averaged GDSF and unpaired neurons to assess the degree of similarity between their waveforms. The corresponding p-value for correlation coefficients was determined using the *t*-distribution.

#### Burst classification

Bursts were classified where the parameters for the **(1)** maximum time between bursting action potentials was set to ≤ 1.5s, the **(2)** minimum burst duration was set to ≥ 0.5s, and **(3)** minimum spikes in a burst was set to ≥ 3 spikes.

### Statistical analysis

A Shapiro-Wilk test was used to determine if spike variables were normally distributed, and to dictate whether a parametric or nonparametric test would be used. A one-way ANOVA or Kruskal Wallis Test was used to assess differences in spike characteristics between culturing conditions followed by Tukey’s multiple comparison test. For comparisons between GDSF and unpaired conditions for bursting parameters, a Mann-Whitney U test was used. A Wilcoxon signed-rank test was used to determine if spike rates and amplitudes increased from baseline at the time of cell-to-cell contact in GDSF conditions for MEA recordings. To compare bursting activity in dish cultures, cells were recorded for 2–6 h after cell culturing. A *one*-way ANOVA was used to assess differences between bursting activity (spikes per burst and IBI) between unpaired neurons with higher or lower levels of neuronal branching and in comparison to paired neurons prior to synapse formation. Paired LPeD1 and VD4 cultured in dishes were recorded from 18 to 20 h following plating, and then a paired t-test was used to compare bursting activity (spikes per burst and IBI) for 1–2 h before and after synapse formation. A one-way ANOVA with Tukey’s multiple comparisons test was used to compare the stimulation paradigm to the bursting activity patterns seen in neurons with higher or lower amounts of neuronal branching. A Holm-Bonferroni correction was applied to all p-values following pairwise t-tests. This includes the p-value for the paired t-test for paired neurons before and after synapse formation. For MEA analysis, when a comparison was made only between the three culturing configurations, Tukey’s honestly significant test was used to account for family wise error instead. For all tests, α was set to 0.05, and two-sided tests were used. All figures used the following significance legend: Not significant (n.s) *P* > 0.05, *P < 0.05, **P < 0.01, ***P < 0.001, and ****P < 0.0001. Standard error of the mean (SEM) is indicated for all figure captions.

## Data Availability

The data that support the findings of the study are available from the corresponding author upon request.
